# Development of a New Measure for Assessing Mentalizing: The Multidimensional Mentalizing Questionnaire (MMQ)

**DOI:** 10.3390/jpm11040305

**Published:** 2021-04-15

**Authors:** Alessio Gori, Alessandro Arcioni, Eleonora Topino, Giuseppe Craparo, Rosapia Lauro Grotto

**Affiliations:** 1 Department of Health Sciences, University of Florence, Via di San Salvi 12, pad. 26, 50135 Firenze, Italy; rosapia.laurogrotto@unifi.it; 2 Department of Education, Languages, Intercultures, Literatures and Psychology (Psychology Section), University of Florence, 50121 Firenze, Italy; alessandro.arcioni25@gmail.com; 3 Department of Human Sciences, LUMSA University of Rome, Via della Traspontina 21, 00193 Rome, Italy; eleonora.topino@gmail.com; 4 Faculty of Human and Social Sciences, UKE—Kore University of Enna, Via Cittadella Universitaria s.n.c., 94100 Enna, Italy; giuseppe.craparo@unikore.it

**Keywords:** mentalization, self-report measure, multilevel model, integration, assessment

## Abstract

This research consists of two studies which aimed to: (1) evaluate the psychometric properties of a new self-report measure for the assessment of mentalizing, the Multidimensional Mentalizing Questionnaire (MMQ); and (2) investigate the ability of the instrument to discriminate between community and clinical populations. A sample of 349 participants (19% male, 81% female; *M_age_* = 38.6, *SD* = 15.3) filled in the MMQ and other self-report measures, in order to assess the factor structure, reliability and some aspects of construct validity of the measure. Then, a clinical sample (*N* = 46; 52% male and 48% female; *M_age_* = 33.33, *SD* = 12.257) and a community one (*N* = 50; 42% male and 58% female; *M_age_* = 38.86, *SD* = 16.008) filled in the MMQ, to assess its clinical sensitivity. The factorial analysis identified six principal dimensions of the measure: reflexivity, ego-strength, relational attunement, relational discomfort, distrust, and emotional dyscontrol. The MMQ showed satisfactory psychometric properties and a theoretically relevant factor structure. Furthermore, significantly greater impairment in mentalizing was found in the clinical sample in respect of the community one. The findings are discussed in terms of clinical implications, emphasizing the usefulness of the MMQ in both research and clinical practice.

## 1. Introduction

Even though mentalizing occurs from a very early age to give some meaning to the environment, an analysis seems to be a hard task because it is a complex construct to identify and enclose within its boundaries. Bateman and Fonagy [1] define mentalizing as “the process by which we make sense of each other and ourselves, implicitly and explicitly, in terms of subjective states and mental processes. A profoundly social construct in the sense that we are attentive to the mental states of those we are with, physically or psychologically” (p. 11). Being a “process”, Allen [2] additionally supports the use of the participle “mentalizing” instead of the noun “mentalization”, in order to emphasize mental activity. Seemingly, mentalizing is rooted in four different areas of psychology: first, cognitive psychology, with the identification of the construct of theory of mind [3], described as a module phylogenetically tasked with processing the others’ mental states. Second, Bion [4] proposed a theory “of the thought” where imagination arises as a response to separation anxiety. The mother, thanks to her *reverie* (i.e., a specific function which allows the mother to feel the infant inside her, and to give shape and words to the infant’s experience), can make sense of the raw material inside the infant (beta elements) to help them create building blocks for their emotional and intellectual development (alpha elements). All this appears to be similar to Fonagy theory. Additionally, French-speaking psychoanalysts provided their contribution in developing the concept of *penseé operatoire* [5]. Today, we may define this concept as a “failure” in the mentalizing process, because it recalls hyper-concretism of thought deprived of its imaginative mental activity. Furthermore, Lecours and Bouchard [6] theorized a development of thought considered today similar to mentalizing. The authors focus on how human beings evolve from libidinal impulses to attribution of meaning. Finally, a relevant contribution was given by Anglo-Saxon psychoanalysis and by the Attachment Theory. Winnicott [7] introduced the concept of maternal *mirroring* within the transitional space set in the dyad with the infant; Bowlby [8] theorized that a positive caregiver-infant interaction favors an attachment system which helps the infant regulate their emotional state. Mayes [9] highlighted that this is easily detected when measuring the arousal levels in infants with differing attachment styles. Recent studies showed how different mentalizing ways are linked to distinct brain region activity and this reflects the actual conception of an environment-dependent construct based on four different polarities: automatic and controlled mentalizing, internal and external mentalizing, self-other mentalizing, and cognitive-affective mentalizing [10]. Controlled mentalizing requires a number of slow and typically verbal skills which demand mental effort, high arousal levels and focused attention; implicit or automatic mentalizing is likely to be based on simple heuristics [11]. Internal mentalizing is referred to as the process focused on thought, feelings, and internal experience of both one’s Self and others, while external mentalizing is referred to as external features and behavior of both one’s Self and others [10]. In normal development, the separation-individuation process drives the infant to differentiate their own experiences from those of others. In this process, reciprocity between mirroring and reflective functioning seemingly plays a crucial role: The implicit perspective-taking is strictly connected to an active giving of meaning and differentiating of the behavior of both one’s Self and others [10]. Cognitive-affective mentalizing has a precursor in the Baron-Cohen dualism, i.e., the theory of mind module and empathy system [3]. Such apparent dichotomy reflects the difference between people who usually tend to interpret their environment through emotions, with the risk of ending in projective identification [12], and those who tend to rationalize their affective experience.

Given the growing interest on mentalizing in mental health, some authors have engaged in the development of assessment methods. These have been developed as experimental-observational tasks, e.g., the Reading the Mind in the Eyes Test [13], narrative-based measures, such as the Reflective Functioning Scale [14], or questionnaires, among which the Mentalization Scale [15], the Mentalization Questionnaire [16], and the Reflective Functioning Questionnaire [17] can be mentioned. Concerning this last type of measures, to the authors’ knowledge, the Italian context includes clinician-report scales, i.e., the Mentalization Imbalances Scale [18], the Modes of Mentalization Scale [19], and a self-report one, i.e., the Italian version of the Reflective Functioning Scale [20]. The latter is an agile eight-item questionnaire with a satisfactory reliability and construct validity which effectively discriminates between borderline personality patients and healthy controls and consists of two subscales giving an evaluation of the respondents’ hypo- and hyper-mentalizing attitudes [20]. However, mentalizing is a multifaceted construct, in which problems can be an expression of imbalance in the different polarities underpin it, while functional levels are an expression of balance between the different dimensions included in mentalizing. Therefore, the present research aims to respond to the need to enhance the framework of the evaluation methods concerning this important construct, by proposing a new self-report questionnaire for the assessment of mentalizing which also explore its subcomponents.

This research consists of two studies. The aim of the first study is to present a new measure for the assessment of mentalizing, the Multidimensional Mentalizing Questionnaire, based on an integrated perspective and inspired by the criteria of brevity, good psychometric properties, and usefulness in therapeutic activity both in the initial stages and during the process. All this, based on these aspects:

1. An assessment procedure of mentalizing based on cognitive-affective, self-other, internal-external, explicit-implicit axes;
2. An integration of both positive and negative mentalizing clusters which express on different polarities.

The aim of the second study is to investigate the clinical sensitivity of the MMQ, by assessing the ability of the measure to discriminate between community and clinical populations. In particular, based on the above-described literature and in recent evidence supporting the role of imbalances in mentalizing and in its dimensions in psychopathology (see [21] for a review), it is supposed to find higher levels of impairment in mentalizing in the clinical sample, also by exploring the subdimensions of the construct.

## 2. Study 1

### 2.1. Materials and Methods

#### 2.1.1. Participants

This study involved a sample of 349 subjects (19.0% male, 81.0% female), with an age ranging from 16 to 20 years (*M* = 38.6, *SD* = 15.3). Most of the subjects were unmarried (59.6% single) and came from central Italy (62.7%). Concerning the professional condition, 32.5% of participants were employees, 31.2% were students and 21.1% were freelance. Furthermore, 129 subjects had a Master’s degree and 131 had a high school diploma. 

#### 2.1.2. Procedure

The 33 items were elaborated to reflect the core aspects of the construct, as described in the Handbook of Mentalizing in Mental Health Practice [12]. This phase has been implemented by organizing focus groups with a pool of researchers and clinical experts, to make this step more effective. The questions were written in order to obtain answers along a five-point continuum, with a Likert scale ranging from 1 = “Not at all” to 5 = “A great deal”. Cohen’s Kappa coefficient was examined to determine inter-rater compliance about the goodness of each item, showing a good concordance (K = 0.80). Participants were randomly recruited through a snowball-like spreading strategy of an anonymous on-line link. They completed the self-report measures together with a demographic questionnaire (sex, age, marital status, profession and degree of study) on the Google Forms platform after they were informed about the aim of the research. Written informed consent was obtained from all subjects. They did not receive any form of compensation for their involvement in the study and were free to leave at any time. The authors assert that all procedures contributing to this work comply with the ethical standards of the relevant national and institutional committees on human experimentation and with the Helsinki Declaration of 1975, as revised in 2008. All procedures involving human subjects/patients were approved by the Ethical Committee of the Integrated Psychodynamic Psychotherapy Institute (IPPI) (ethical approval number 002/2020).

#### 2.1.3. Measures

##### The Multidimensional Mentalizing Questionnaire (MMQ)

The Multidimensional Mentalizing Questionnaire (MMQ) is a self-report measure that consists of 33 items, covering the different core aspects the construct on four different axes: (1) Cognitive–affective; (2) self–other; (3) outside–inside; and (4) explicit–implicit. Items were reviewed for clarity and to avoid ambiguity or double negatives. Response format was on a five-point Likert scale from 1 = “Not at all” to 5 = “A great deal”.

##### 20-Item Toronto Alexithymia Scale (TAS-20)

The 20-Item Toronto Alexithymia Scale (TAS-20) [22,23] is a 20-item self-report scale designed to assess the level of alexithymia. Each item is rated on a five-point scale ranging from 1 (strongly disagree) to 5 (strongly agree), measuring three main dimensions: (1) difficulty in identifying feelings and distinguishing between feelings and bodily sensations in emotional activation, (2) difficulty in the verbal expression of emotions, and (3) externally oriented thinking. In this study the Italian version of Bressi and colleagues [24] was used and showed a good internal consistency with a Cronbach’s alpha of 0.84.

##### Barratt Impulsiveness Scale (BIS-11)

The Barratt Impulsiveness Scale (BIS-11) [25] is a 30-item self-repot measure designed to assess impulsiveness. Each item is rated on a four-point Likert scale ranging from 1 (rarely/never) to 4 (almost always/always), measuring six first-order factors grouped into three second-order factors: (1) Attentional impulsiveness, composed by attention and cognitive instability first-order factors; (2) motor impulsiveness, composed by motor and perseverance first-order factors; and (3) non-planning impulsiveness, composed by complexity and self-control first-order factors. In the present study the Italian version of Fossati and colleagues [26] was used, showing a satisfactory internal consistency (α = 0.75).

##### Rosenberg Self-Esteem Scale (RSES)

The Rosenberg Self-Esteem Scale (RSES) [27] is a 10-item self-report questionnaire designed to measure global self-esteem. Each item is rated on a four-point scale ranging from strongly agree to strongly disagree. In this study the Italian version of Prezza and colleagues [28] was used, showing good internal consistency (α = 0.89).

##### General Self-Efficacy Scale (GSE)

The General Self-Efficacy Scale (GSE) [29] is a 10-item self-report questionnaire designed to measure self-efficacy. Each item is rated on a four-point Likert scale ranging from 1 (not at all true for me) to 4 (very true for me). In this study the Italian version of Sibilia, Schwarzer, and Jerusalem [30] was used and showed a good internal consistency with a Cronbach’s alpha of 0.91.

#### Psychological Treatment Inventory—Attachment Styles Scale (PTI-ASS)

The Psychological Treatment Inventory-Attachment Styles Scale (PTI-ASS) [31] is a 22-item self-report scale designed to assess the quality of adult attachment and is a section of the Psychological Treatment Inventory [32]. Each item is rated on a five-point Likert scale ranging from 1 (Not at All) to 5 (A Great Deal) and evaluates attachment style between in secure, preoccupied, avoidant or unresolved. The subscales’ Cronbach α in the current study were of 0.80, 0.84, 0.78, and 0.68, respectively.

##### Italian Ten Item Personality Inventory (I-TIPI)

The Ten Item Personality Inventory (TIPI) [33] is a 10-item self-report scale designed to assess personality traits according to the Big Five model [34]. Each item is rated on a seven-point Likert scale ranging from 0 (Disagree strongly) to 7 (Agree strongly), which evaluates 5 dimensions: extraversion, agreeableness, conscientiousness, emotional stability and openness. In this study, the Italian Ten Item Personality Inventory (I-TIPI) of Di Fabio, Gori and Giannini [35] was used, with subscales’ Cronbach α of 0.89, 0.70, 0.76, 0.83 and 0.79, respectively.

##### Insight Orientation Scale (IOS)

The Insight Orientation Scale (IOS) [36] is a seven-item self-report measure designed to assessing some of the characteristics of insight, including behaviors, feelings and opinions about this construct. Each item is rated on a five-point Likert scale ranging from 1 (not at all) to 5 (a great deal), focused on seven core aspects of insight: level of consciousness, problem solving, restructuring (behavior change), awareness, complexity (abstraction, depth), surprise, and self-reflectiveness (thoughtfulness). In the present sample, the scale showed a Cronbach α of 0.76.

#### 2.1.4. Data Analysis

Data were analyzed with the SPSS software (IBM-SPSS 25.0 version, IBM, Armonk, NY, USA) for Windows and MPlus Version 8.1 [37]. Descriptive statistics were calculated. An exploratory factor analysis (EFA) with a principal axis factoring extraction method (Promax rotation with Kaiser normalization) was performed in order to verify the factor structure of the Multidimensional Mentalizing-Q. Then, the factor model was tested through a confirmatory factor analysis (CFA), considering the following indices: (1) the model chi-square (*χ*^2^), indicating a good model fit when the probability value is nonsignificant [38]; (2) the root mean square error of approximation (RMSEA), with accepted values ≤ 0.08 [39]; (3) the Tucker Lewis index (TLI), for which Kline [40] considers reasonable values ≥ 0.90; (4) the Comparative Fit Index (CFI), for which reasonable values were ≥0.90 [40]; (5) the standardized root mean square residual (SRMR), with recommended values ≤ 0.08 [41]. Reliability of both, the scale and its factors, was calculated using the Cronbach’s alpha coefficient. Finally, in order to assess some aspects of construct validity, Pearson’s correlation was carried out between the MMQ subscales and the other investigated variables.

### 2.2. Results

Descriptive statistics for the sample were reported in Table 1, while means and standard deviations were showed for each item of the Multidimensional Mentalizing-Q in Table 2. 

Skewness and kurtosis of the MMQ total score were between −1 and +1 (0.04 and −0.06, respectively) and the mean value of the total score was 111.58 (*SD* = 11.24). 

**Figure 1:**
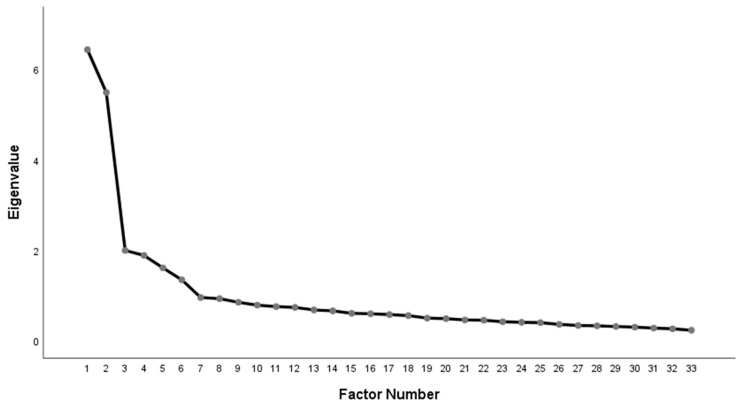
Scree plot.

Results of an exploratory factorial analysis (EFA) using the principal axis factoring method (Promax rotation with Kaiser normalization) showed a factor structure with six principal dimensions, which combined explained 56.9% of the total variance (eigenvalue = 1.35). The first factor accounted for 19.51% of the variance and was made up of nine items indicating reflexivity; the second one accounted for 16.64 of variance and consisted of six items related to ego-strength; the third one accounted for 6.05% of variance and included five items describing relational attunement; the forth one accounted for 5.72% of variance and was composed by five items referring to relational discomfort; finally, the remaining two factors (both of four items) accounted the 4.88% and 4.08% of variance and indicated distrust and emotional dyscontrol, respectively (see Figure 1). The factor correlation matrix showed a prominent inter-correlation among factor scales, indicating that the questionnaire subscales measured several dimensions of mentalizing, relatively distinct from each other (see Table 3).

Concerning the confirmatory factor analysis (CFA), the goodness-of-fit indices indicated a satisfactory fit of the six-factor model. Indeed, although the Model Chi-Square was significant (*χ*^2^ = 134.88, *p* < 0.001), the other indices showed satisfactory values (RMSEA = 0.053; TLI = 0.90; CFI = 0.90; SRMR = 0.067). Then, the reliability of the scale was calculated using the Cronbach’s alpha coefficient and indicated a good level of internal consistency for both the total scale (α = 0.75) and the subscales (factor 1, α = 0.89; factor 2, α = 0.81; factor 3, α = 0.82; factor 4, α = 0.76; factor 5, α = 0.74; factor 6, α = 0.72). Finally, Pearson’s correlation was carried out to assess convergent and discriminant validity (see Table 4).

The MMQ subscales showed significant correlations with most of the measures used to assess construct validity.

## 3. Study 2

### 3.1. Materials and Methods

#### 3.1.1. Participants and Procedure

This study involved a community sample and a clinical one. The latter consisted of 46 individuals (52% male and 48% female), with an age ranging from 18 to 62 years (*M_age_* = 33.33, *SD* = 12.257). They were recruited in various private clinical settings and received a diagnosis in line with the International Classification of Diseases-11th Edition [42]: Schizophrenia or other primary psychotic disorders (10.9%), mood disorders (28.3%), anxiety or fear-related disorders (19.6%), obsessive-compulsive or related disorders (19.6%) and personality disorders and related traits (21.7%). The community sample consists of 50 individuals (42% male and 58% female), with a mean age of 38.86 (SD = 16.008; ranging from 20 to 76). All participants completed a paper-pencil questionnaire, and written informed consent was obtained from all subjects. Privacy and anonymity were guaranteed. The authors assert that all procedures contributing to this work comply with the ethical standards of the relevant national and institutional committees on human experimentation and with the Helsinki Declaration of 1975, as revised in 2008. All procedures involving human subjects/patients were approved by the Ethical Committee of the Integrated Psychodynamic Psychotherapy Institute (IPPI) (ethical approval number 002/2020).

#### 3.1.2. Measures

##### The Multidimensional Mentalizing–Q (MMQ)

The *Multidimensional Mentalizing–Q* (MMQ) was used to assess the level of mentalizing, considering the multifaced nature of the construct. Indeed, this 33-item self-report measure permits a multidimensional assessment, with scores on the positive (Reflexivity, Ego-strength and Relational Attunement) and negative (Relational discomfort, Distrust and Emotional dyscontrol) subscales, as well as an overall MMQ score, by summing all the items after having reversed those included in the negative subscales.

#### 3.1.3. Data Analysis

Data were analyzed with the SPSS software (IBM-SPSS 25.0 version, IBM, Armonk, NY, USA) for Windows and MPlus Version 8.1 [37]. The MMQ scores and those of its subscales were compared in the community and clinical samples, by using an independent samples *t* test. A two-tailed *p* value of less than 0.05 was considered as statistically significant.

### 3.2. Results

The independent-samples t-test showed significant differences in the MMQ total score and its subscales between the community and clinical samples, except for the factors of relational attunement and emotional dyscontrol (see Table 5).

## 4. General Discussion

**Figure 2:**
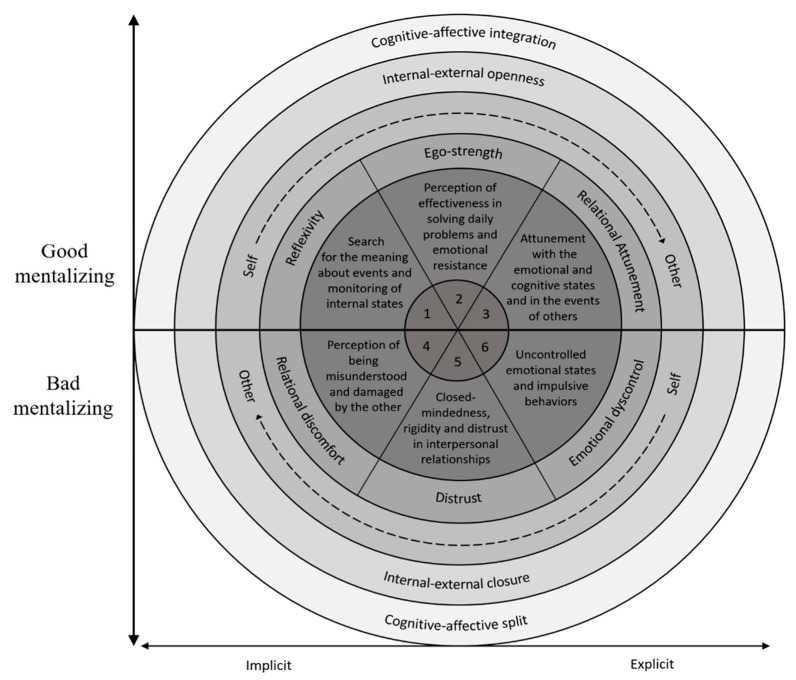
Integrated and multilevel model of mentalizing.

The aim of this research was the development of a new measure for the assessment of mentalizing, the Multidimensional Mentalizing Questionnaire (MMQ), and to evaluate its psychometric properties and clinical sensitivity, also illustrating and supporting a new integrated and multilevel model of mentalizing (see Figure 2). This conceptualization, in fact, moves on four axes that intertwine and explicate in the different factors found in MMQ: cognitive-affective, self-other, outside-inside, explicit-implicit. Good levels of mentalizing presuppose the balance of these polarities, which must be used flexibly according to the needs and the environment [12]. This, therefore, implies an alternation of implicit (fast insight) and explicit (metacognition) modalities with harmony between cognitive and affective aspects, without one persistently dominating the other. Furthermore, mentalizing guides the interpersonal relationships, through the ability to proactively focus on internal elements while maintaining a functional awareness of the external world and with the oscillation between the perspective of the self and that of the other: it requires the ability to see “ourselves from the outside” and “others from the inside” [43] (p. 347). The imbalance of these dimensions could be seen as a core aspect in mental illness [12] and is, therefore, important to pay attention to them during assessment phases, evaluating their combinations in setting treatment programs to improve outcomes. 

The MMQ showed satisfactory psychometric properties, with a clear and theoretically relevant factor structure, an adequate internal consistency and good construct validity. Mentalizing is a broad and multifaceted concept that encompasses and combines multiple constructs involved in treating others and ourselves as social agents [44]. As a reflection of this, the exploratory factor analysis (EFA) indicates a six-factor structure, also confirmed by the confirmatory factor analysis (CFA): the first three subscales (reflexivity, ego-strength, and relational attunement) are “positive” and highlight functional components of mentalizing, while the last three (relational discomfort, distrust, and emotional dyscontrol) are the “negative” opposites, referring to failures and distortions. Reflexivity appears to be strictly linked meta-cognition, introspection, and critical thinking [45], indicating a propensity towards the search for a deep understanding of one’s experiences: It manifests itself with interest and curiosity for the exploration of one’s mental states and with the desire to analyze behaviors and events. It is conceptually opposed to emotional dyscontrol, that refers to the difficulty to manage own affective states and to a tendency to impulsiveness. Dysregulated activations undermine the ability to mentalize [44]: Indeed, hyperarousal states can lead to a temporary “blindness” linked to the prevalence of limbic activity over cortical one, hindering the ability to minding the mind, which is closely related to the prefrontal cortex elaborations [46]. The second positive factor, ego-strength, is a key component of resilience [47] and concerns the perception to be able to face everyday problems with an emotional resistance to stress and frustrations: “Ego strength is the foundation from which a person can move forward into the environment” (ibidem, p. 21). It favors the metabolization of painful experiences without these damaging the self, keeping realistic trust and a sense of efficacy [44]. Its opposite is distrust, described as an attitude of closed-mindedness, distrust in relationships and tendency to have a black or white view of the world. This leads to the immersion of the subject in sturdy vicious circles where the conceptions of himself as fragile and the other as threatening are repeatedly confirmed, with a strong mistrust regarding external and unknown experiences that instead could instead expand and enrich the social understanding of the individual: this absence of trust, therefore, impairs the ability to change [48]. Finally, relational attunement indicates the ability to tune in the emotional and cognitive states of the others and deeply understand their experiences. It is a component of empathy [49], a necessary side of mentalizing [44] that focuses most on understanding the others, acquiring their perspective with a subject-object state matching [50]. It finds its opposite in relational discomfort, characterized by interpersonal difficulties and the perception of being misunderstood and damaged by others: this determines relational insecurity, fear of abandonment and pessimistic closure in one’s own self. It could also be seen as a typical manifestation of the non-mentalizing state of psychic equivalence, in which thoughts are experienced as facts, without the modulation of mental processes of higher levels and, therefore, with unshakable strength and intensity: one’s own painful mental state, in other words, means that others are bad [51]. Thus, in the present model, the positive subdimensions and negative ones (each with good values of internal consistency) could be conceptualized as opposite poles of a continuum of good-bad mentalizing. The former, as shown by the correlations, are all significantly and positively associated with secure attachment, openness, and self-efficacy, contrasting instead with alexithymia and impulsiveness. The negative poles, on the other hand, present a diametrically opposed framework. This could be read in light of the clinical research suggesting the role of attachment patterns in being facilitators or inhibitors of mentalizing [1,44,52]. In secure one, the mental states are discovered through mirroring and contingent interactions with the caregiver [53]: their reaction to the communicative manifestations of the child lead the latter to understand the effects of their behavior and develop a perception of themselves as effective [54]. Furthermore, this also allows to increase and confident disposition towards the exploration, identification and expression of one’s mental states, favoring greater openness to experience. In this way, the subject who grew up in an environment capable of satisfying basic human needs will develop adaptive social mentalities [55] and this will make them able to use the information capacity of their feelings [56] in the interpersonal sphere promoting positive and healthy relationships, also having a greater effectiveness in the management of conflicts [44]. On the other hand, insecure attachment patterns are associated with difficulties in understanding and regulating emotions, due to unresolved past events that continue to keep a trace in the present [57]. The lack of awareness of one’s feelings limits access to effective regulatory strategies, thus resulting in impulsive and destructive reactions [58]. All this negatively impacts the subject in many spheres of his life, with a tendency to instability, affective lability, suspiciousness, relational insecurity, and social avoidance. 

Consistently, results showed significantly lower mentalizing skills in the clinical sample than in the community one, also demonstrating the clinical sensitivity of the MMQ. This is in line with the scientific literature highlighting the association between imbalances of mentalizing and psychopathology (see [21] for a review), and this was further confirmed by the exploration of the MMQ sub-dimensions. Concerning the positive ones, indeed, the clinical sample showed significant lower levels in Reflexivity and Ego-strength than the community one. Such findings are consistent with previous research showing severe deficits in metacognition, that “capitalizes on the reflexive nature of consciousness” [59] (p. 50), in several kind of psychopathology, such as personality disorders, schizophrenia and bipolar disorders (see [60] for a review). This implies an abstract and generic reflexive modality that fail to explain related thoughts, feelings or intentions of one’s self or of others, with a deficit on critical thinking manifesting itself with the tendency to have rigid beliefs considered indisputably true about themselves and others [61]. Furthermore, the reflexive process is related to the strength of the ego and its organizing skills, acquired by means of the containing relationship with the caregiver [62]: the awareness of being an individual having a mind and the consequent reflexive functioning develops within the secure attachment, strongly associated with important aspects for the constitution of the self, including cognitive competence, exploratory ability, ego resilience and ego control, and frustration tolerance [52]. On the contrary, attachment insecurity is a major contributor to mental disorders [63]. It is characterized by a caregiver failure in responding to the child’s emotional and physical needs and this leads to compromised mentalizing abilities and epistemic mistrust [53,59]. Especially, previous research [64] showed that the mentalizing process is the mental activity linking the internal working models to the perception of strain in interpersonal contact and problems in the interpersonal functioning, which is compromised in many forms of psychopathology. This is reflected in the findings of the present research, in the exploration of negative mentalizing subdimensions: indeed, results showed significantly higher levels in relational discomfort and distrust in the clinical sample than in the community one. 

This research presents some limitation that should be identified and discussed. Firstly, the differences between the different psychopathologies in the total levels of mentalizing or in the different subcomponents were not investigated, also due to the size of the clinical sample. This could be of great interest for future research, also using the MMQ to explore mentalizing from a multidimensional perspective. Furthermore, data were collected by the use of only self-reported measures, that could be subject to upward and social desirability bias. Finally, the MMQ requires a self-evaluation of aspects of which one might not have a full awareness. In future research, the use of a multimodal approach (e.g., with the integration of structured interview) could permit to have a more complete and accurate assessments, overcoming these issues.

## 5. Conclusions

Important implications can be drawn from the current study. The MMQ showed good psychometric properties, and the rapid and easy administration of the measure allow a comprehensive assessment of mentalizing, also considering its dimensionality. Moreover, this research highlighted the clinical sensitivity of the MMQ, further supporting the association between the imbalances of mentalizing and psychopathology. Such findings may contribute to underline the centrality of the mentalizing construct in different forms of pathology. Indeed, previous research showed that neural injuries may affect mentalization abilities in a body-specific manner [65], and that targeted physiotherapy can improve such mentalizing deficits, possibly in association with physical improvements [66]. Furthermore, the integration of ours results with evidences in the field of neuro-psychology may also offer a new reading key to favor a broader understanding of the psychiatric aspects of the neuro-cognitive relationship between mentalizing and cortico-spinal excitability [67]. On that basis, this study may favor insights on possible homologies between mentalization-related consequences of the psychiatric conditions and previous evidences on neurological injuries. Therefore, the MMQ can be usefully adopted in both research and clinical practice.: the theoretical framework of mentalizing proposed here, the integrated and multilevel model of mentalizing, can provide important suggestions for psychotherapy and treatments. In conclusion, the MMQ may be a valuable self-report for repeated measurement of client status over the course of therapy, favoring tailored interventions and supporting clinical research.

## Figures and Tables

**Table 1:** Demographics variables of the sample (*n* = 349).

Characteristics	*M* ± *SD*	*n*	%
Age	38.56 ± 15.27		
Sex			
Male		78	19.04
Female		319	80.96
Provenience			
Northern Italy		79	20.05
Central Italy		247	62.69
Southern Italy		68	17.26
Marital Status			
Single		235	59.64
Married		119	30.20
Separated		18	4.57
Divorced		17	4.31
Widowed		5	1.27
Professional condition			
Unemployed		6	1.52
Freelance		83	21.07
Employee		128	32.49
Trader		10	2.54
Housewife		22	5.58
Student		123	31.22
Retired		16	4.06
Artisan		6	1.52
Study degree			
Elementary school (5 years)		4	1.02
Middle School diploma		36	9.14
High School diploma		131	33.25
University degree		75	19.04
Master’s degree		129	32.74
Post-Lauream Specialization		19	4.82

**Table 2:** Factor structure of the Multidimensional Mentalizing Questionnaire and means and standard deviations of the items.

Item	F1	F2	F3	F4	F5	F6	M(SD)
1. I often try to explain what is happening to me *^a^* *Provo spesso a darmi delle spiegazioni su ciò che mi accade*	0.80						4.11 (0.92)
16. I ponder over what happens to me *^a^* *Rifletto su quello che mi succede*	0.79						2.83 (1.25)
18. I often think about why things happen *^a^* *Rifletto spesso sul perché delle cose*	0.78						2.84 (1.36)
32. I’m keen on understanding why certain things happen to me *^a^* *M’interessa capire perché certe cose mi accadono*	0.73						3.87 (0.91)
10. I’m interested in understanding my mental processes *^a^* *M’interessa comprendere i miei processi mentali*	0.67						3.77 (1.02)
17. I find beneficial to analyse my behaviour *^a^* *Trovo beneficio ad analizzare il mio comportamento*	0.62						4.26 (0.86)
31. I am a thoughtful person *^a^* *Sono una persona riflessiva*	0.53						2.57 (1.24)
8. I am able to reflect on my behaviours *^a^* *Sono in grado di riflettere sui miei comportamenti*	0.47						4.18 (0.76)
6. Understanding what others feel is crucial in understanding their actions *^a^* *Capire ciò che gli altri provano è importante per comprendere le loro azioni*	0.44						1.91 (1.07)
30. I am able to cope with difficult situations *^a^* *Sono in grado di affrontare situazioni difficili*		0.79					4.20 (1.01)
25. I am able to bear the emotional load of stressful situations *^a^* *Sono in grado di sopportare il carico emotivo delle situazioni stressanti*		0.76					3.46 (0.98)
24. I am able to sort out difficult problems when life presents those to me *^a^* *Sono in grado di risolvere problemi anche complessi che la vita mi mette davanti*		0.71					2.51 (1.17)
11. I can tolerate frustrations of daily life *^a^* *Sono in grado di tollerare le frustrazioni della vita di tutti i giorni*		0.68					2.61 (1.17)
22. I can usually adapt myself to different contexts with no difficulties *^a^* *In generale so adattarmi a diversi contesti senza difficoltà*		0.54					4.06 (0.93)
26. When I feel an intense emotion, I can control it *^a^* *Quando provo un’emozione forte riesco a controllarla*		0.44					2.54 (1.27)
28. I can easily attune to other people’s thinking *^a^* *Riesco a sintonizzarmi facilmente sul pensiero altrui*			0.83				4.23 (0.88)
5. I can tune in other other people’s mental states *^a^* *Riesco a sintonizzarmi sugli stati mentali degli altri*			0.80				4.00 (0.98)
14. I’m able to empathize with others when they tell me something *^a^* *Mi immedesimo negli altri quando mi raccontano qualcosa*			0.66				4.14 (0.97)
4. I’m able to get the deepest aspects of people around me *^a^* *Riesco a cogliere gli aspetti più profondi delle persone a me vicine*			0.61				2.46 (1.31)
21. I am sensitive to what happens to others *^a^* *Sono sensibile a quello che accade agli altri*			0.49				2.61 (1.16)
12. Others don’t understand me *^a^* *Gli altri non mi capiscono*				0.70			4.13 (0.85)
9. Relationships with other people prevent me from being myself *^a^* *Le relazioni con gli altri mi impediscono di essere me stesso*				0.68			3.90 (1.01)
27. People abandon me *^a^* *Le persone mi abbandonano*				0.59			3.39 (1.09)
15. I am afraid to open up with other people *^a^* *Ho paura ad aprirmi con gli altri*				0.56			3.77 (0.91)
33. Some people are the cause of my problems *^a^* *Alcune persone sono la causa dei miei problemi*				0.39			3.53 (1.09)
13. It’s better to beware of others *^a^* *È meglio stare attenti agli altri*					0.76		3.06 (1.08)
29. It’s better to beware of strangers *^a^* *Bisogna ben guardarsi dalle persone che non si conoscono*					0.73		1.97 (1.17)
20. I don’t trust others *^a^* *Non mi fido degli altri*					0.56		3.76 (1.00)
19. For me things are either white or black *^a^* *Per me le cose sono o bianche o nere*					0.42		2.76 (1.21)
2. I am an impulsive person *^a^* *Sono una persona impulsiva*						0.65	3.89 (0.91)
7. I sometimes feel like I am losing control of my emotions *^a^* *A volte ho la sensazione di perdere il controllo delle mie emozioni*						0.59	4.02 (0.98)
3. I sometimes experience mood swings I can’t control *^a^* *Talvolta ho degli sbalzi di umore che non riesco a controllare*						0.56	4.09 (1.04)
23. It happens to me to have conflicting emotions *^a^* *Mi capita di provare emozioni contrastanti*						0.53	2.73 (1.36)

Note: F1 = reflexivity (riflessività; α = 0.89); F2 = ego-strength (adattamento; α = 0.81); F3 = relational attunement (sintonizzazione relazionale; α = 0.82); F4 = relational discomfort (disagio relazionale; α = 0.76); F5 = distrust (sfiducia; α = 0.74); F6 = emotional dyscontrol (discotrollo emotivo; α = 0.72). Italics indicate the Italian version of the Multidimensional Mentalizing-Q. *^a^* English translation of the Multidimensional Mentalizing-Q.

**Table 3:** Descriptive statistics and factor correlation matrix.

Factors	M	SD	Skewness	Kurtosis	Factor Correlation Matrix *					
					1	2	3	4	5	6
1. Reflexivity	36.63	5.32	−0.55	−0.17	1					
2. Ego-strength	21.61	4.31	−0.57	0.75	0.10	1				
3. Relational attunement	19.60	3.58	−0.57	0.25	0.55	0.23	1			
4. Relational discomfort	11.67	4.32	0.80	0.34	0.09	−0.52	−0.08	1		
5. Distrust	10.44	3.63	0.34	−0.32	−0.04	−0.35	−0.19	0.47	1	
6. Emotional Dyscontrol	11.63	3.65	0.08	−0.70	0.11	−0.37	0.06	0.38	0.29	1

* Extraction Method: Principal axis factoring. Rotation method: Promax with Kaiser Normalization.

**Table 4:** Correlation matrix.

	1	2	3	4	5	6	7	8	9	10	11	12	13	14	15	16	17	18	19	20	21	22	23	24	25
1. MMQ (F1)	1																								
2. MMQ (F2)	0.228 **	1																							
3. MMQ (F3)	0.556 **	0.163 **	1																						
4. MMQ (F4)	−0.106*	−0.431 **	−0.068	1																					
5. MMQ (F5)	−0.168 **	−0.310 **	−0.131 **	0.509 **	1																				
6. MMQ (F6)	0.019	−0.389 **	0.034	0.411 **	0.339 **	1																			
7. TAS20	−0.491 **	−0.353 **	−0.327 **	0.535 **	0.423 **	0.432 **	1																		
8. TAS20 (F1)	−0.196 **	−0.435 **	−0.082	0.538 **	0.367 **	0.553 **	0.815 **	1																	
9. TAS20 (F2)	−0.337 **	−0.240 **	−0.219 **	0.488 **	0.353 **	0.303 **	0.800 **	0.528 **	1																
10. TAS20 (F3)	−0.632 **	−0.073	−0.491 **	0.144 **	0.223 **	0.052	0.629 **	0.199 **	0.308 **	1															
11. BIS11	−0.362 **	−0.194 **	−0.217 **	0.233 **	0.173 **	0.419 **	0.462 **	0.362 **	0.261 **	0.418 **	1														
12. BIS11 (F1)	−0.263 **	−0.240 **	−0.137 **	0.335 **	0.246 **	0.345 **	0.487 **	0.414 **	0.357 **	0.317 **	0.716 **	1													
13. BIS11 (F2)	−0.099 *	0.037	0.013	0.097	0.095	0.362 **	0.245 **	0.207 **	0.138 **	0.202 **	0.726 **	0.321 **	1												
14. BIS11 (F3)	−0.422 **	−0.226 **	−0.328 **	0.123*	0.075	0.263 **	0.333 **	0.224 **	0.129*	0.409 **	0.817 **	0.379 **	0.367 **	1											
15. RSES	0.080	0.479 **	0.046	−0.595 **	−0.352 **	−0.410 **	−0.451 **	−0.526 **	−0.387 **	−0.051	−0.214 **	−0.244 **	−0.068	−0.176 **	1										
16. GSE	0.230 **	0.686 **	0.195 **	−0.370 **	−0.207 **	−0.251 **	−0.364 **	−0.439 **	−0.231 **	−0.103*	−0.204 **	−0.200 **	0.061	−0.293 **	0.586 **	1									
17. PTI (F1)	0.253 **	0.354 **	0.223 **	−0.367 **	−0.157 **	−0.090	−0.355 **	−0.293 **	−0.326 **	−0.178 **	−0.158 **	−0.151 **	−0.020	−0.177 **	0.365 **	0.358 **	1								
18. PTI (F2)	0.085	−0.344 **	0.093	0.495 **	0.324 **	0.419 **	0.281 **	0.396 **	0.258 **	−0.072	0.079	0.194 **	0.051	−0.033	−0.481 **	−0.286 **	−0.222 **	1							
19. PTI (F3)	−0.017	0.111 *	0.059	0.102*	0.067	−0.009	0.084	0.025	0.036	0.141 **	0.088	−0.004	0.175 **	0.033	0.022	0.142 **	−0.283 **	−0.073	1						
20. PTI (F4)	−0.014	−0.179 **	0.010	0.381 **	0.152 **	0.292 **	0.212 **	0.273 **	0.146 **	0.026	0.221 **	0.149 **	0.168 **	0.181 **	−0.311 **	−0.153 **	−0.397 **	0.324 **	0.169 **	1					
21. I-TIPI (F1)	0.105 *	0.253 **	0.097	−0.366 **	−0.157 **	0.099*	−0.231 **	−0.164 **	−0.280 **	−0.081	0.106*	0.020	0.195 **	0.035	0.278 **	0.299 **	0.236 **	−0.165 **	−0.043	−0.034	1				
22. I-TIPI (F2)	0.049	0.179 **	0.117*	−0.187 **	−0.161 **	−0.334 **	−0.204 **	−0.200 **	−0.163 **	−0.083	−0.169 **	−0.133 **	−0.174 **	−0.088	0.164 **	0.139 **	0.147 **	−0.090	−0.048	−0.120*	−0.033	1			
23. I-TIPI (F3)	0.109 *	0.280 **	0.116*	−0.262 **	−0.021	−0.286 **	−0.233 **	−0.252 **	−0.164 **	−0.087	−0.425 **	−0.305 **	−0.250 **	−0.392 **	0.300 **	0.264 **	0.160 **	−0.116*	0.037	−0.210 **	−0.071	0.284 **	1		
24. I-TIPI (F4)	0.022	0.494 **	−0.015	−0.373 **	−0.224 **	−0.476 **	−0.353 **	−0.490 **	−0.233 **	−0.007	−0.247 **	−0.306 **	−0.112*	−0.160 **	0.484 **	0.469 **	0.226 **	−0.396 **	0.083	−0.202 **	0.014	0.263 **	0.291 **	1	
25. I-TIPI (F5)	0.274 **	0.344 **	0.205 **	−0.224 **	−0.257 **	−0.064	−0.229 **	−0.138 **	−0.162 **	−0.230 **	−0.019	−0.101*	0.163 **	−0.091	0.137 **	0.250 **	0.169 **	−0.101*	0.109*	−0.122*	0.251 **	0.046	−0.050	0.149 **	1
26. IOS	0.514 **	0.507 **	0.408 **	−0.324 **	−0.177 **	−0.213 **	−0.504 **	−0.418 **	−0.359 **	−0.351 **	−0.359 **	−0.307 **	−0.108 *	−0.378 **	0.407 **	0.578 **	0.361 **	−0.191 **	0.047	−0.122*	0.145 **	0.158 **	0.247 **	0.354 **	0.229 **

** Correlation is significant at the 0.01 level (2-tailed). * Correlation is significant at the 0.05 level (2-tailed). MMQ = Multidimensional Mentalizing Questionnaire; MMQ (F1) = reflexivity; MMQ (F2) = ego-strength; MMQ (F3) = relational attunement; MMQ (F4) = relational discomfort; MMQ (F5) = distrust; MMQ (F6) = emotional dyscontrol; TAS20= 20-item Toronto Alexithymia Scale; TAS20 (F1)= difficulty identifying feelings and distinguishing between feelings and bodily sensations in emotional activation; TAS20 (F2) = difficulty in the verbal expression of emotions; TAS20 (F3): externally oriented thinking; BIS11= Barratt Impulsiveness Scale 11; BIS11 (F1) = attentional impulsiveness; BIS11 (F2) = motor impulsiveness; BIS11 (F3) = non-planning impulsiveness; RSES = Rosenberg self-esteem scale; GSE = general self-efficacy scale; PTI (F1) = secure attachment; PTI (F2) = preoccupied attachment; PTI (F3) = avoidant attachment; PTI (F4) = unresolved attachment; I-TIPI (F1) = extroversion; I-TIPI (F2) = agreeableness; I-TIPI (F3) = conscientiousness; I-TIPI (F4) = emotional stability; I-TIPI (F5) = openness; IOS= insight orientation scale.

**Table 5:** Independent samples t-test results for Multidimensional Mentalizing Questionnaire and its subscales’ scores between the community and clinical samples.

	Community Sample (*n* = 50)		Clinical Sample (*n* = 46)		*t*	*df*	*p*	95% Confidence Interval of the Difference	
	*M*	*SD*	*M*	*SD*				Lower	Upper
MMQ total score	113.08	11.127	105.70	10.673	3.314	94	0.001	2.960	11.809
Reflexivity	37.20	4.567	32.85	5.428	4.262	94	0.001	2.325	6.380
Ego-strength	21.30	4.432	17.15	5.011	4.303	94	0.001	2.234	6.062
Relational attunement	18.92	4.174	18.83	3.542	0.118	94	0.906	−1.482	1.670
Relational discomfort	11.90	3.955	14.30	4.857	−2.669	94	0.009	−4.193	−0.615
Distrust	11.54	3.655	15.02	3.073	−5.029	94	0.001	−4.856	−2.107
Emotional Dyscontrol	12.22	3.710	11.80	4.539	0.489	87.101	0.626	−1.275	2.106

## Data Availability

The data presented in this study are available on request from the corresponding author. The data are not publicly available due to privacy reasons.
